# The person in the disabled body: a perspective on culture and personhood from the margins

**DOI:** 10.1186/s12939-016-0437-2

**Published:** 2016-09-15

**Authors:** Maayan Agmon, Amalia Sa’ar, Tal Araten-Bergman

**Affiliations:** 1 The Cheryl Spencer Department of Nursing, Faculty of Social Welfare and Health Sciences, University of Haifa, 199 Aba Khoushy Ave, Mount Carmel, Haifa, 3498838 Israel; 2 Department of Anthropology, University of Haifa, 199 Aba Khoushy Ave, Mount Carmel, Haifa, 3498838 Israel; 3 School of Social Work, Faculty of Social Welfare and Health Sciences, University of Haifa, 199 Aba Khoushy Ave, Mount Carmel, Haifa, 3498838 Israel

**Keywords:** Personhood, Health, Disability, Marginalized, Inequality, Disparity, Cultural, Ethnography, Israel, Discrimination

## Abstract

**Background:**

Persons with disabilities (PWD) are one of the most marginalized groups in Western societies. These inequalities are manifested through various disadvantages in the psychosocial, cultural, and economic domains. Inspired by the World Health Organization's holistic conceptualization of disability, the present study examines the relation between the body and personhood in Israeli culture, through cases of newly diagnosed adults with disability.

**Method:**

Participant observation at a rehabilitation daycare center was carried out for a period of two years. The analysis is based on field notes recorded during these observations, including interviews with individuals with disabilities, their family members, and service providers.

**Results:**

The analysis reveals the agonizing experience of individuals who have become disabled in adulthood, who undergo symbolic diminution and social exclusion after their former acceptance as whole and normative persons. This ongoing multifaceted process includes infantilization, denial of their sexuality/sensuality, transgression of gender boundaries, and their construction as categorically different from the "healthy" people around them. At the same time, the analysis also demonstrates the ways in which daily routine at the daycare center also complicates the normative healthy-disabled binary, indicating a continuum on which attendees may attempt to reposition themselves.

**Conclusions:**

This paper aims to make a dual contribution. We draw on anthropological understandings of“person” as a holistic category to resurrect the personhood of individuals with disabilities, as a correction tothe overwhelming tendency to reduce their humanity to their physical injury. We likewise reverse theanalytical gaze by using these individuals' experiences to understand the normative, culture-bound perception of “healthy” persons. We thus highlight Israeli culture's conditioning of normative personhood on having a perfect body, and its concomitant construction of individuals with physical disabilities as lesser persons. By opting to bring back the person into the disabled body, we aim to facilitate a less stigmatized outlook on disability and to create an opportunity for caregivers, researchers, and healthcare professionals to view disabled persons as whole and complex human beings.

## Background

The aim of this study is to identify ingrained, culturally bound assumptions inherent the treatment of people with disabilities, and how, through renewed dialogue, a shift can take place in their treatment away from a singular focus on their physical condition and towards a more holistic focus on their personhood.

Person with disabilities (PWD) are one of the most marginalized groups in Western societies. International studies have demonstrated a persistent link between disability status and socioeconomic and health disparities. For instance, people with disability are more likely to live in poverty [1, 2], demonstrate lower levels of workforce participation and educational attainment [3, 4]; they may also face violence and discrimination related to their disability and have difficulties accessing appropriate health care [5]. Moreover, despite the enactment of national and international legislation aimed at ensuring equal opportunities for people with and without disabilities and the prohibition of discrimination on the basis of disability, these patterns of inequality remain significant. Acknowledging the reasons for these social gaps [6], scholars have called for a more holistic understanding of the processes and mechanism by which atypical bodies are translated into reduced social standing and forced into a distinct sense of personhood in a given culture [7–10].

In recent years, research on the body in general [11–13] and on the disabled body in particular, has assumed a prominent place in the health and social science literature [14, 15]. Despite wide variation in topics and interpretations, three underlying assumptions characterize this literature. First, disability is a typically distressing experience, accompanied by stigmatization that often leads to socio-economic disparities and social exclusion [16]. Secondly, the disabled body is increasingly seen as a stopping point on a continuum of health, rather than as a counter-category of “able bodied” [7, 14]. Thirdly, disability is socially constructed insofar as there are cross-cultural variations in its definition, experience and management [17, 18].

This paper explores the experiences of newly diagnosed Israelis with disability. In keeping with the holistic and culturally embedded approach to disability, we take the focus off the afflicted body, the physical and mental components of disability, the stigma, and the practical aspects of its management, and look at the impact of the physical disability of these individuals on their personhood. Disability studies have long argued against the reduction of persons with disability to their disabled body, with its inevitable compromise of their integrity [19]. We endorse this position and take it one step further by arguing that the experiences of newly disabled persons can illumine the articulation of personhood in their culture more generally. Having lived to adulthood as able bodied people before becoming disabled, these individuals offer a unique perspective on both normative and aberrant personhood. We use the term “person” as an analytical and normative concept that allows us to regard the participants as part of a larger social context [20].

While we reject the traditional focus on physical disabilities and choose instead to concentrate on the person in the disabled body, the former remains important to us empirically and analytically. The influential work of Pierre Bourdieu, deflected the focus from social facts to the way such facts were created and recreated “from organism to embodiment” [21]. The concept of “habitus”, for example, has advanced our capacity to undo the antinomy between structure and agency, and to show how humans internalize social structures which they then reproduce through practice [22]. Body “hexis” [23] likewise articulates the ways cultural knowledge is simultaneously stored in and produced through the body.

Concomitantly, the term “embodiment” directs us to trace the ways complex interactions involving social knowledge, internalized beliefs, identities and bodily practices are objectified into size, shape and appearance, then made subjective again through practice [24–26]. It allows us to identify processes whereby abstract cultural knowledge is incorporated and transformed into concrete behavior, sensation and perception. It also serves to grasp and articulate the dialectics between the physical body and the political body [27]. It thus “encourages reanalysis of existing data and suggests new questions for empirical research” [24].

Recently, concepts such as “embodiment”, “habitus” or the idea of “the body as social capital” have been gaining ascendancy, including outside anthropological and cultural studies. Disability studies, as already noted, are increasingly looking at the impact of social relationships on health and illness, and at the impact of the latter on social identities and social standings [28–30]. Similarly, the bio-social model of the World Health Organization’s (WHO 2001) International Classification of Functioning, Disability and Health (ICF), acknowledges that biology and society are entwined in a dialectical relationship that constructs disability as the result of negative interactions between an individual’s impairment and the physical-social environment in which he or she lives [31, 32]. The ICF conceptualization goes beyond medical impairment and calls for an all-inclusive view of the person as a holistic entity, placing the person’s body and experiences at the center of inquiry. Health, as portrayed by the ICF, is a dynamic interaction between the individual’s functioning, resources and socio-cultural context. Instead of defining health as the direct opposite of disability, these two constructs are placed on a continuum [31, 33]. Consequently, health and disability are viewed as a holistic human experience, in which biological, individual, social and environmental aspects are invariably integrated. Moreover, this conceptualization highlights the important role of professionals and policy agencies in shaping the ways individuals with disabilities experience their lives, and the crucial role of bureaucratic procedures in facilitating or hindering their social inclusion. Lastly, the ICF recognizes that disability is embedded in complex socio-political contexts, so that its meanings and experiences are also mediated by social variables such as gender, family status, work capacity, social class and ethnicity [18, 34]. Hence, the ICF framework may serve as a conceptual framework for exploring equity and social justice. According to this framework, the person’s experience is shaped by interacting with social processes and structures, which are also impacted by power, social place and time. Our study accords with the ICF holistic perspective and humanistic philosophy, but adds a cultural angle, as we explore the relation between perceptions of disabled bodies and culturally specific notions of personhood. We also go beyond the discourse of functioning, disability and health, by using the experiences of subjects whose bodies have become disabled to understand the notion of personhood in their culture more generally.

In Israeli culture the healthy body is a key component of normal personhood [35]. The forming of the Jewish nation has centered on the project of forming a new Jew, with sturdy muscles [36] and an upright body, to replace the presumed feeble and disabled body of the Jew in exile. The subjects with disabilities presented in this study, in their unintended deviation from the ideal of “the chosen body” [27] shed]  light not only on the local cultural understanding of a healthy body, but also on what it means to be a “worthy” person.     

## Methods

The materials for this study were collected using qualitative anthropological methods that include participant observation and in-depth open interviews. Participant observation is designed to encompass the full range of practical and discursive knowledge, thus enabling the researcher to explore the actors' point of view as historically situated and context-bound [23, 37]. Between 2002 and 2004, Ma'ayan Agmon conducted participant observation at the center. She attended the center three days a week and participated in their events, such as holidays and birthdays celebrations. She kept a field diary documenting the various activities, conversations and her own reflections, eventually writing an ethnography of the center. The present paper is based on observations, interviews and field notes taken throughout the two-year period. Analysis of the data involves three steps, the first step was coding the material, the second step involved organizing the material into themes by the research team (MA, AS), and the third step involved interpretation of the themes by using continuous reflexivity [38]. Reflexivity was achieved by paying careful attention to, and discussing together, the emotional, cognitive and moral reactions of the first author in the field, including her newfound doubts about her self-perception as “healthy.”

Setting: The research was conducted at a rehabilitation daycare center in a city at the center of Israel, run by a large voluntary society. It caters to 35 attendees, aged 45 through 60 years (average 52). Most suffered brain damage and were transferred to the daycare center following a period of rehabilitation in a hospital. The attendees are considered “young disabled”, according to the definition of the Israel National Insurance Institute. At the time of the study four professionals worked at the center: a nurse who was also the director, an occupational therapist, a physical therapist and a volunteer psychologist. All four spent relatively few hours weekly at the center, leaving most of its administration and operation to thirty volunteers with no formal training or experience in rehabilitation.

Services at the center included transport to and from the attendees’ homes, cooked meals, physical and occupational therapy, and cultural activities such as handicrafts and lectures on literature, Bible and history, as well as basic training in computer skills and the Internet. Two series of group meetings, one for the attendees and one for the volunteers, were facilitated by the physical therapist and the psychologist. The volunteers eagerly offered the attendees companionship and assistance.

## Results

The findings were organized according to themes. Following the qualitative methodology of anthropological tradition, each theme will be supported by participant’s quotations.

*“They think that a disabled person is also wrong in the head”–The social projection of injured body to personhood*

In the course of the fieldwork at the center, definitions that at first appeared self-evident came under question and emerged as more complex. One seemingly clear-cut classification was that of disabled attendees and able-bodied caregivers. The “disabled” were those who had a visible disability and who had been referred to the center on account of any number of diagnoses provided by the medical establishment. All others were assumed to be caregivers by default. The taken-for-granted attribution of disability to visible bodily defects came up naturally in the introduction of the center to visitors, including the first author, who was unwittingly led to classify the person seated in a wheelchair or going about with a cane and a splint as a patient, and the one walking upright on his or her own two feet as a caregiver.

A more subtle observation, however, revealed that the rigid division between disabled attendees and able-bodied caregivers did not go unchallenged. Rafi, a man in his fifties, paralyzed in the lower part of his body and mobile by means of a powered wheelchair, is a case in point. Having initially arrived at the center as an attendee, he soon assumed the position of its secretary:

*I was here a month as an attendee. I went to the director and told her I was leaving. This place wasn’t worth fifty shekels a day. Most of the volunteers here are crap. One day they come, the next they don’t. I really like to paint* (referring to the drawing classes), *but it isn’t worth the money. The director didn’t want me to leave, so she sent me to a computer course. I learned Word, and since then I’ve been working here as secretary, volunteering for 300 shekels a month.*

Another example of the local dichotomy of disabled attendees and able-bodied caregivers is Tehiya, who was introduced to the first author as a volunteer. Tehiya walked on her own two feet and was very well groomed, wearing stylish clothes and makeup. She even showed up for the meetings of the care givers team. It took the first author a long time to notice that the other volunteers never approached her for help or cooperation, as if there was an unspoken agreement on the subject. Tehiya also suffered several bouts of hypoglycemia to the point of fainting, when an ambulance was needed to take her away. Asked why Tehiya did not come to the center as an attendee, but received the symbolic payment of volunteer, the director replied:

*Tehiya is not disabled. She has lots of health problems, but you can’t insist that she come as an attendee, and she can’t be asked to stop coming because that is what keeps her going in life. The organization we belong to does good deeds, and this is one of them.*

While Tehiya and Rafi may represent exceptions, their cases serve as useful markers of a more consistent, though subtle, rule. Participant observation at the center revealed that despite the formal construction of the disabled and the able-bodied as mutually exclusive categories, the actors’ embodied knowledge implied a continuum of positions. In some cases such knowledge indeed facilitated creative negotiations, as exemplified by Rafi and Tehyia.

*“Since the event I’ve become half a person”–Embodiment of the social projection to the self*

The relationships that were formed at the center were largely focused on the body. Talking about the body established a new discourse on the person, revealing that the disabled body created an impaired, childlike, dependent self that lacked elements of sexuality and was deprived of its previous functions, such as in the area of gender.

The injured body was the main subject of discussion during the first encounter between the caregivers and the attendees. Attendees spent a considerable number of hours every day managing their bodies and tending it in various ways. The volunteers and staff likewise frequently undertook tasks concerned with bodily needs: accompanying attendees to the restroom, serving food, performing physical and occupational therapy, and preparing handicrafts. Attitudes to the body were expressed in several ways: the attendees referred to themselves in terms of “before” and “after” the injury, comparing themselves with each other and with the world of the able-bodied, on a scale of independence–dependence.

Attendees frequently expressed frustration with their lack of control over their bodies. They used poignant metaphors of a broken or malfunctioning body that brought into sharp relief the cultural emphasis on control over the body as an essential component of adult personhood:

Aryeh: *Since the event I’ve been half a person—half is saying too much: a living dead person. On one side you see how he’s dead. The tonus goes higher and higher, the hand is completely paralyzed, the leg is splinted. Dr. Zaiger told me on my last visit to Leowinstein, that if it doesn’t improve we’ll have to give injections to release the tonus.*

Gadi: *I have no stability in my body. My body doesn’t listen to me. People see me and they think that any second now I’m going to fall over and whoops— they want to grab me. It’s like I’m drunk and going to topple over any minute. It’s not as bad as it looks, but my body doesn’t listen to me.*

Zeev: *I’ve got this weird thing that I cry and laugh without control. Suddenly something small happens and I begin to cry like a baby and it’s out of control. I can’t stop it. Same with laughing. I laugh and because of the ataxia the laughter sounds really loud and people look at me and think that I’m a bit wrong in the head.*

The underlying assumption that the visibly damaged body entails cognitive disability came up frequently in the attendees’ narratives. For example, they related how doctors would choose to direct the explanation about the injury to their spouses and not to them, even when they themselves were present. They were well aware of these situations, which recurred in different variations in their daily lives, making it difficult to go out alone.

In the therapy group the attendees were asked to draw their bodies as part of an exercise to build a bridge to their unconscious. After completing the drawing, they were asked to talk about it. The drawing below exemplifies the theme of lack of control, a feeling so powerful that it surprised even Adiva, the woman who drew it:
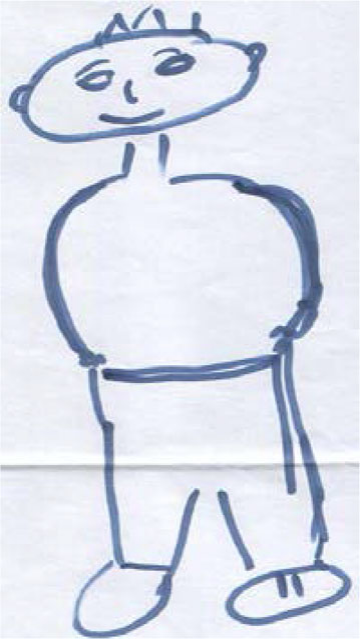

*First of all, what amazed me is that I didn’t draw one hand, my paralyzed hand. Apparently I have internalized the fact that it’s not going to work again. A while ago I asked the doctor what he thought about it and he said that in his opinion the hand wouldn’t work anymore. And it hurts me to discover that I’ve come to terms with it, as if this hand isn’t part of my body.*

The theme of control and lack of control over the body was particularly prominent in attendees’ interactions with the medical establishment and medical personnel. Apparently, upon experiencing new and unfamiliar feelings, attendees approached the medical personnel, who established an initial sense of control over the condition by the very naming of it. Attendees showed minimal resistance to what the physicians had to say, and even when they tried to resist were easily silenced by their relatives and acquaintances. They and their relatives tended to treat the opinions voiced by members of the medical establishment about the nature of their injury as highly authoritative.

Yishai explained:

*The doctor said the thing I suffer from, it’s a kind of depression. It has a direct connection to brain damage. A lot of people suffer from it. Obviously, something got damaged in the brain and in the areas connected to depression. He gave me pills and said it was for life. I took the pills–what difference does it make, one pill more, one pill less. I personally don’t think I’m in depression. The social worker thinks the same as the doctor. She talked to my wife about it, and began to do her head in with it and said it was normal and that there are stages of mourning. My wife came to talk to me about it. I don’t believe in this dime psychology, but if it does my wife and the doctor good I don’t mind taking another pill.*

Attendees tended to be very conscious of the unusual state of their bodies, and they said that this awareness was intensified by the attitudes of those around them. Sometimes the attitudes that they absorbed most strongly illuminated the symbolic burden carried by their exceptional body, as well as other underlying assumptions regarding disability that encompass a person with an injured body. The attendees reported that sometimes they took advantage of these preconceived ideas, by allowing themselves to behave in unacceptable ways or by falsely pretending to be unable to perform certain tasks. But above all, they resented these assumptions because they prevented them from participating in social life along with those who looked, and were perceived as, healthy. As Rafi, the attendee in a wheelchair who volunteered as secretary, described it:

*People see a person in a wheelchair and at once think that there’s something wrong with him. I’ll tell you a story that you won’t believe. One day someone or other from the municipality came in here looking for Zefira. I told her that I was the secretary here and asked if I could help her. She ignored me and went to Tami to ask her. She thought that I was wrong in the head or something. I told her, “What, you don’t think a man in a wheelchair can tell you the truth?” But by now I’m used to this attitude and sometimes I make good use of it. For example, when I go to the Town Hall I start yelling straight away. I don’t care if they think I’m a nutcase. The main thing is that they give me what I want right away.*

Yishai expressed similar sentiments about being treated as if he were incapable of doing anything:

*The volunteers here think that we can’t do anything because we’re handicapped. For example, they hand everything to us, and if I go near the hot kettle they start shouting straight off as if I were a three-year-old. At home I make myself coffee without any problem. I can be on my own for hours sometimes. But here they leap up to do things for me.*

By underestimating disabled persons’ abilities, and by doing things for them that they can do for themselves, the staff harm their self-confidence. The attendees perceive and convey this dependence as something negative and undesirable in a society that nurtures independence, hence reinforcing and legitimizing their exclusion. From the interactions between the attendees and the medical establishment, which are the predominant interactions in the construction of disability, many hidden messages emerge, at times more meaningful than overt messages. These covert messages take the form of ignoring the disabled person and promoting explanation-giving and decision-making between the doctor and the family, while omitting the client themselves.

*“They’re like children to me”-Construction of the person with disability as childlike and asexual*

Sexuality and gender as dominant components of adult personhood, were always present within the walls of the center. Prominent motifs in the construction of these issues were fracture and continuity, although attendees and their spouses differed in the expression of these motifs. While the former bore an overwhelming sense of rupture, the latter actually inclined to continuity in matters concerning their disabled spouse’s sexuality.

Denial of sexuality by those whose bodies had betrayed them was ubiquitous at the center. The relations between attendees and caregivers evinced consistent disregard for accepted codes of interaction between men and women, as verbal and physical gestures, which outside the center would probably be interpreted as sexual harassment or condescension, were completely normative. It was very common, for example, to see women volunteers addressing the male attendees using terms of endearment, such as “honey” and “baby,” accompanied by a physical pat on the head. When questioned about this behavior, the women, most of whom were religious, therefore forbidden any physical contact with unrelated adult men, typically alluded in their answers to the men’s pathetic and miserable state: *“They’re like children to me.”* Similarly, when male attendees complimented female volunteers on their appearance, the women did not seem offended by such seeming breaches of decorum; they simply dismissed them offhandedly, claiming that this behavior was like that of children and could be overlooked.

*“We are celebrating like kids”–Celebrations as a social projections*

Another common practice of infantilizing was the importance allotted to ceremonies and special events in the center’s routine. These included birthdays, holidays and special events, which in Israel are usually celebrated by children at school and kindergarten, but not by adults at workplaces. For example, Issachar’s birthday was celebrated on a weekday. All the participants were seated around a table laden with goodies, such as chocolates, cookies, soda and cake. Most participants there suffered from diabetes and usually made an effort to avoid these foods. The birthday started with some birthday songs, followed by eating the food. The birthday party was described as an excuse to ignore their diet:

Issachar: *Even the doctor said that every once in a while, at special events, we are allowed to deviate from our diet and eat some candies.*

The participants were asked if they marked their birthdays that way before they arrived at the day center:

David: *Believe me, I didn’t even know what date my birthday was until I arrived here. When they asked me, it took me a couple of minutes to remember.*

Adiva: *We usually celebrated within the family. It wasn’t a big deal. We ate a cake. We did it mostly for the children.*

Ester: *I was a pre-school teacher, and the way we celebrate here reminds me of those days. It looks exactly the same with the candies and the songs. The only difference is that in pre-school the birthday kid sat on a chair and we lifted him up in the air. We don’t do that here. Ever since I started coming here I have brought a cake and celebrated, pretending it’s a real celebration.*

Janet: *What can I tell you— it’s nice to celebrate. With me, since my husband died and I have only one child, we didn’t really celebrate. Here I really feel everyone’s happy with me. It’s important, that attention.*

Other holidays were celebrated using a format that is more accepted among children. After a party on Purim (a Jewish holiday where people wear costumes), the participants described their feelings:

Uri: *I wanted to go home, and my wife wanted to stay. I don’t like all these parties, and it’s always crowded and you can’t move. If you want to go to the bathroom you have to move all the people sitting next to you. I don’t like these things, and my wife forces me.*

Adiva: *I don’t like these kinds of celebrations either, but the volunteers invest a lot of effort in it, so we have to come and thank them. We have to show our appreciation.*

The argument that the participants did not fully identify with the nature of the event, but cooperated to satisfy the volunteers, was repeated several times during the fieldwork.

*“I was a living man. Now I’m a broken reed”*-*Gendered experiences of disability*

Differences between men and women stood out sharply in all aspects of sexual and gender self-perception. The subjects of conversation between men and women were fairly stereotypical and replete with blatant symbols of sexuality. For example, at three meetings of the therapy group, the conversation centered on driving and vehicles, but quickly shifted into relations between men and women prior to the injury. In these conversations the women were silent, only requesting a change of subject after several meetings:

Yissachar turns to Aryeh: *You see, soon you’ll have a van, and you’ll be able to drive around town like a man.*

Aryeh: *What man, what kind of a man? The days are over when I can go around like a man. Once there were women tourists in town and then I really did go around like a man–in every sense of the word.*

Group leader: *What d’you mean?*

Aryeh: *What d’you mean, “What d’you mean”? Either you get it or you don’t. I had a fabulous woman here. We had the time of our lives for years. I was a living man. Now I’m a broken reed.*

Janet: *A woman while you were married?*

Aryeh: *Yes. A wife is a wife and a woman is to have a good time with.*

David: *There’s nothing like a wife. A wife knew you before the injury–that’s a different kind of knowing. She knows what I was before the injury*

[David’s speech is met with the men laughing and the women looking embarrassed.]

David: *Memories, memories. There’s a lot to remember. You can never do it like it was…*

Yissachar: *If you did it like you did it once, you’d fall out of bed.*

The metaphor of mobility and the inability to drive a vehicle and thus move freely in the public sphere, came up time after time among the men, in juxtaposition to their boasting about sexual relations with different women. They constantly lamented their injured body, which limited them and their sexual activity, and reminisced about their past sexual prowess.

Unlike the men, who persistently bemoaned their difficulty in sexual functioning, the women ignored this subject entirely and repeatedly talked about their distress at being unable to perform house work and care for others. The problem they constantly complained about was their inability to do things the way they had, such as entertaining relatives and running the home properly, which they perceived as their sole sphere. Janet said:

*The hardest thing for me is that I can’t prepare things for my children and my grandchildren like I did before-me, who used to do a hundred things all at once. In the mornings I’d go to women at home and give them a pedicure, then I’d work all day at the factory and go home. You should’ve seen my house–always clean, always food…. Where’s all that now? I can barely manage to have a cake for visitors when they come.*

When the subject of sexual relations came up, and difficulty in sexual functioning compared with prior to the injury, all the women, without exception, spoke of their sorrow concerning the pain their husbands had to endure because of their impaired functioning, an issue that did not arise with the male attendees in respect of their wives. A picture of passivity was painted when the women were asked about sexual relations before and after the injury, as they expressed a desire to satisfy their partner, but did not perceive the lack of sexual passion as a significant loss for their own quality of life:

Yaffa: *Me? What am I worth? Obviously, my husband, who is still young, will go and look at other women. It’s natural.*

Adina: *If it was the other way round, then what would you do?*

Yaffa: *Not me. But women and men are not the same thing in these things. Women have other needs.*

In sum, regarding sexuality the men focused on their own sense of fracture and loss, while the women emphasized the fracture in the experiences of the able-bodied husband, who now had to live with a disabled woman. While they were worried that their sexually frustrated husbands might leave them, none of them lamented the loss of their own sexual pleasure.

“*He was hardly involved before either”-Spousal re*latio*nships in the context of disability*

Attendees—men and women alike—described brain damage as a serious breaking point in spousal relations. They noted a change in their spousal relations as a result of their becoming dependent and less functional. This perception stood in stark contrast to the way their spouses described the relationships. Typically, the spouses depicted a continuous pattern of relationship, only with the problems intensified. In three different conversations, held in different situations, attendees’ spouses noted how matters continued just as prior to the injury. The pattern of relations had not changed; it was simply augmented by the disabled body and the need to treat it. Aryeh’s wife substantiated this theme:

*“He was always that way, hardly involved, hardly any contact with the children. Only before the injury he would be out all day long. We hardly saw him at home. Now he sits in the living room, so it’s all more extreme.”*

Attendees” references to sexuality and gender contained elements of both rupture and continuity. At the center, de-sexualizing the attendees’ bodies entailed their infantilization, so that they became exposed to behaviors common between adults and children. In their spousal relations the partner with disability highlighted the element of crisis, while the able-bodied partner stressed continuity, explaining that the bodily injury had complicated the familiar pattern, but without making it radically different. Regarding the ability to engage in sexual relations, the men underlined the element of fracture and incompetence, which they associated with their impaired ability to drive. The women described their sexuality as passive even before the event, and highlighted the fracture in their ability to perform their domestic functions.

Another example of the gender differences at the center, was the presents given on birthdays, which symbolized the perception of continuity of gender among the attendees. Despite feeling the limitations caused by the injury and complaining about it on other occasions, they continued to receive gifts according to the perception that characterized them before the injury. For example, men usually got a pen, women a body lotion. This kind of presents was particularly interesting since some of the participants had limited ability to use their hands.

Integration of the themes included in this ethnography reveals that people with disability undergo symbolic infantilizing and de-sexualization, which feeds back into their construction as “half persons”, or defective human beings. As discussed below, this reduction in the category of “persons” reflects the perception prevalent in Israeli culture that a normal person is one whose body is healthy and functioning properly and independently. The failure of people with an injured body to live up to this standard therefore entails depletion of their humanity.

The message that the attendees were lesser persons was communicated through a series of symbolic dichotomies. A basic distinction between healthy and disabled was reinforced through the symbolic treatment of the latter as children and their construction as asexual. It was likewise expressed in attendees’ continuous lamenting their inability to fulfill their appropriate gender assignments, and through their poignant sense of disjunction between their life before and after the injury. While participant observation revealed that these distinctions did not go unchallenged, the message they communicated was unmistakable and their cumulative effect powerful and oppressive.

## Discussion

### Disability as a window on normative personhood

The main findings of this research expose a central facet of inequity and social exclusion in Israeli society. The findings reveal that the adult person in Jewish Israeli culture is expected to be physically flawless, to function independently and to be in full control. S/he is perceived as different from the child and as having the option of various courses of possible action. The unblemished body and independent adult person are one, and when the body is injured, the visible damage is associated with a damaged person. Analysis likewise shows that the person is established in a cultural space replete with dichotomies (able-bodied vs. disabled, adults vs. children, women vs. men, flawless vs. damaged), with little room left for ambiguous positions. Accordingly, the injured body represents a retreat to a childish, pre-sexual and dependent state, with a limited ability to make choices.

Our finding that the injured body serves as a primary reference for all the actors at the rehabilitation center illustrates Bourdieu's theoretical concept of body hexis and the dialectics between formal and practical knowledge [23]. We have shown that the vernacular classification of attendees and caregivers draws almost exclusively on the presence or absence of visible disabilities. This reflects the official knowledge regarding health and disability as mutually exclusive categories. Yet beyond passive embodiments of pathology, we have also shown that attendees' bodies comprise a major site of cultural production. As such, they at once reaffirm and potentially avert the naturalization of social structures and symbolic hierarchies. While for the most part attendees tend to incorporate the stigma into their most intimate experiences of subjectivity, the same body may also provide the instrument for their agentive reclaiming of social personhood.

The perception of disabled persons as possessing childlike characteristics was reflected in their being engaged in handicrafts suitable for children, in the manner of physical touch and addressing them in endearing language as if they were asexual, or in the common tendency of doctors to ignore them when providing medical explanations, addressing their relatives or friends instead. This tendency is rooted in Israeli culture more generally, as corroborated in the literature which highlights the importance of the whole, healthy, strong body as a ticket to the Israeli collective [27, 36]. Attendees in general tended to accept their framing as “half persons,” albeit with lamentation, particularly concerning their inability to uphold normative gender performances. They tended to reminisce about their former pre-injury life, and expressed a strong sense of rupture. Lastly, besides these effects, the statements of attendees and caregivers alike also revealed an implied juxtaposition of physical injury and cognitive damage, although such an association was not necessarily supported by clinical evidence.

This social construction of individuals with disabilities draws on three interrelated classifications: that produced by the psychological discipline, that produced by the biomedical establishment and that which is part of Israeli culture more generally. These three distinct cultural discourses share a perspective of the universe organized across a grid of rigid dichotomies [9, 39, 40]. In the biomedical discourse these dichotomies are expressed by medical classifications such as “normal” vs. “abnormal.” As expressed by Rafi, one of the attendees: “*My doctor speaks to my wife and not to me. He thinks that the disabled body is also a disabled mind.”* In the psychological discourse they are expressed in strict distinctions between men and women, children and adults, and again, normal and abnormal [41]. The Israeli cultural discourse, lastly, has filled these categories with a social content that may well be even more rigid, especially regarding the body and its physical performance. For instance, Israel has the highest rate of abortions of imperfect embryos in the world and a flourishing industry of pregnancy check-ups [35, 42, 43]. This emphasis on the unblemished healthy body, which recurs in various situations all through the individual’s life span [36], emanates partly from the collective memory of the Holocaust [44], but also from the Zionist ideology of the “new Jew” [45]. Although they are not identical and in fact draw on distinct historical legacies, the three cultural discourses–the biomedical, the psychological and the general Israeli–effectively reinforce each other in creating a sense that healthy and injured persons are ultimate opposites, and that injury to the body automatically also impairs the person’s mental, sexual and social faculties. As a result, injured individuals experience multi-faceted exclusion, far beyond their physical incapacities.

The ethnography of the rehabilitation daycare center shows that although the physically injured body is the obvious center of attention of attendees and caregivers alike, what is actually communicated and negotiated among them is the value of the former as members of society, and whether they may still count as “full persons.” Attendees feel trapped in their injured bodies; they mostly tend to submit to their redefinition as depleted persons, and with it to the social exclusion that accompanies it. Still, they are aware of the gaps between what their bodies *can* and *should* do, and between the ways in which they as injured *persons* can and should act. Occasionally, they also manipulate these gaps, as with Rafi’s making a scene at the Town Hall. They may also attempt to broaden their autonomy, as with Yishai’s demanding to make his own coffee, or challenge their exclusion head-on, as in the case of Rafi redefining his role at the daycare center from attendee to secretary. Undoubtedly, this entire communication is indirect. It therefore does not comprise a direct challenge to the hegemonic cultural logic, as represented by the medical and psychological authorities, where the disabled body inevitably disables the person as a whole. It does, however, open a window to the otherwise unmarked category of the “healthy” person, showing that it too is culturally specific.

This paper aims to make several contributions: on the clinical level it illustrates that the caregiver is a cultural mediator, whose behavior is invariably guided by cultural assumptions about the qualities of the healthy or the sick person. These assumptions are inadvertently shared with the person being treated, thereby contributing to their exclusion from society and feelings of isolation. Understanding the impact of cultural assumptions on the perception and treatment of patients with disability concurs with the increasing calls to make healthcare services culturally sensitive. In this case, cultural sensitivity assumes the dual sense of adapting professional and popular attitudes in order to skirt the most demeaning aspect of stigma: the depletion of the person; and of acknowledging that the construction of health and disability alike are always embedded in webs of cultural meaning and social relations. We believe that acquaintance with the overt and covert assumptions regarding the person can change these attitudes, therefore contributing to more sensitive treatment of people with physical injury. This position clearly resonates with the ICF’s commitment to incorporate social relations into the definition of disability, but also takes it a step farther by incorporating a distinctly cultural perspective. National and international policies should acknowledge this link and therefore translate it into enforceable mechanisms promoting the human rights realization and equal participation of people with disabilities.

On a more theoretical level, our choice of the term “person” is pertinent to the attempt to fine-tune the incorporation of a cultural perspective into the medical and psychological professions, a longstanding project in itself. This choice reinforces the call in the anthropological literature to shift from the construct of “self”, to the broader construct of “person” [46, 47]. In psychological and psychiatric discourses, “self” tends to be dominated by pathologies. It also tends to be discussed in isolation from the full, embedded context in which actual people spend their lives. The construct of “person”, by contrast, is more holistic and less a prisoner of biomedical and psychological discourses, therefore lending itself to a more humanistic discussion. Besides helping us to neutralize the psychological load inherent in “self”, the more expansive anthropological formulation of “person” also allows us to see individuals through their relationships, and through the array of forces and social structures that affect their lives [46, 47].

Lastly, on the political level, persons with disability articulating their unique voice and using it as a platform for minority rights activism, have been the driving force of disability studies [46, 48]. In documenting the routine experiences of life with disability and using them as a lens to observe the broader meanings of *life* after disability, this study corresponds with this discourse directly. However, we take issue with the tendency still lingering in some disability studies to retain the traditional dichotomy of the healthy vs. the disabled. Our study strives to regard disabled individuals first and foremost as persons, who occupy a particular point on a continuum of health and wellbeing.

## Conclusion

We hope that by bringing to the fore the culture-bound assumptions that inform the treatment of people with disabilities, this paper will promote a shift from regarding them diminutively as those whose physical injury has disqualified them also mentally and socially, to seeing them as whole, complex persons. Moreover, the study may shed some light on the process and mechanisms by which inequity is evolving. Our findings illustrate that inequity can be identified at two levels of analysis, societal and individual. At the societal level the findings illuminate the different ways in which the atypical body is translated into a reduced social standing, which may result in the marginalization and social exclusion of people with disabilities, as well as active discrimination against them.  The second level of analysis, namely the individual perspective, demonstrates how the impairment of individuals' capacity to perform physical tasks is mirrored in their social interactions with professionals and in their overall institutional experiences, creating unnecessary negative outcomes. Problematizing normative perceptions and a renewed dialogue are likely to be instructive in addressing the array of forces acting on caregivers and patients alike. In addition, defining the subjects as "persons" encourages both researchers and caregivers to view people with disabilities in a wider and more comprehensive context – past, present and future – and as bearers of unique agency. Lastly, using the “person” as an analytic category, allows a broader perspective of the power-structure-agency complex that shapes the lives of people with disabilities and defines the contours of their transformation and adaptation. Such an open and fruitful dialogue and expanded perception of the “person” releases persons with disabilities from the cage of stigmatization, universal assumptions and cultural constructions.

## Abbreviations

ICF: International classification of functioning, disability and health
PWD: Persons with disabilities
WHO: World Health Organization
